# Proceedings of EMMJ6 Medical Journals Conference held at Shiraz Iran (February 18-20, 2015)

**DOI:** 10.12669/pjms.313.8112

**Published:** 2015

**Authors:** Shaukat Ali Jawaid

Shiraz (Iran): Eastern Mediterranean Association of Medical Editors (EMAME) organized its EMMJ6 Medical Journals Conference here from February 18-20, 2015 which attracted distinguished medical editors from the region. It was organized in collaboration with Iranian Society of Medical Editors, Shiraz University of Medical Sciences, Islamic World Science Citation Center, WHO EMRO, Pakistan Association of Medical Editors (PAME) and Asia Pacific Association of Medical Editors (APAME). Some of the unique features of this conference were innovative topics for plenary sessions and panel discussions, honouring the distinguished editors from the region, institution of Young Investigator’s Award and Workshops on topics which were never covered in the past.

**Figure:**
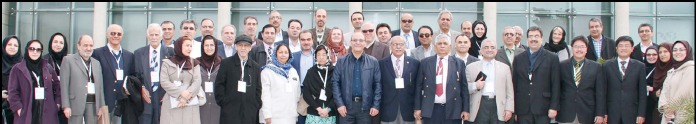
Some of the delegates who attended the EMMJ6 Medical Journals conference held at Shiraz Iran from February 18-20, 2015 photographed with the members of the organizing committee and Executive Committee members of EMAME.

Prof. Farhad Handjani, President Elect of EMAME was the chairperson of the organizing committee while another distinguished editor Dr. Farrokh Habibzadeh former President of World Association of Medical Editors (WAME) who is also Chief Editor of International Journal of Environment and Occupational Medicine was the chairperson of the scientific committee who did a commendable job. Despite the fact that the organizers got just two months time but both Prof. Farhad and Dr. Farrokh Habibzadeh along with their teams were able to put up a good show and had made excellent arrangements for the conference held at the National Library Convention Center.

In the inaugural session after a brief welcome address by Dr. Mohammad Hadi Imanieh Chancellor of SUMS, Dr. Jehane Tawilah WHO Representative in Iran read the message from the WHO Regional Director who asked the researchers and editors to focus on priority areas. Statistical data, it was sated, is extremely vital for planning. One cannot plan for health services if there is no data. Biomedical journals, it was further stated are important to publish research and disseminate the outcome of research. If out come of research does not reach the audience and planners, it will have no impact. Editors, the WHO Director General’s message said, are guarantors of publications. They must practice peer review system and uphold professional ethics and standards in research publications.

WHO it was further stated continues to support medical journals in the region. It has established IMEMR the regional database and at present about six hundred biomedical journals from the region are covered in this index. WHO EMRO is collaborating with EMAME to ensure professional capacity building of the Editors and its positive results are now visible. There are some challenges which will always exist and it was up to EMAME to tackle these challenges in collaboration with the National Association of Medical Editors in different countries. EMAME the WHO Director General hoped will play an important role in bridging the gap between research publications besides promoting and supporting good publication practices.

A video recorded message from **Dr. Maqbool H. Jafary** President of EMAME who could not attend due to health reasons, was shown to the participants. He pointed out that while medical editors and their national associations have been quite active in Iran and Pakistan, there has been no academic activity in other countries in the region which needs to be looked into. He hoped that under the dynamic leadership of the new President Prof. Farhad Handjani, EMAME will make tremendous progress.

EMAME also set a unique example of honouring distinguished editors of biomedical journals from the region. Those who were selected for this honour included Dr. Karim Vessal a distinguished Editor from Iran who was associated with Iranian Journal of Medical Sciences for a long time and at present is the Chief Editor of Iranian Journal of Radiology. Second editor who was honoured was Dr. Maqbool H. Jafary from Pakistan who was Editor-in-Chief of Pakistan Journal of Medical Sciences.. He has to his credit a large number of publications besides being the author of numerous books. Third editor to be honoured was Dr. Ahmad Said El Morsy from Egypt who is Editor of Egyptian Journal of Histopathology. All these three distinguished Medical Editors were presented special mementoes by Jane Nicolson from WHO EMRO and Prof. Mohammad Javed Dehghani President of Islamic World Science Citation Center (ISC). Dr. Karim Vessal and Ahmad Said El Morsy were present on this occasion and received the mementoes themselves while President PAME Dr. Fatema Jawad received the memento on behalf of Dr. Maqbool H.Jafary.

**Open access increases visibility, ensures more readership more citations and high Impact Factor-M. Reza Ghane**

**Figure:**
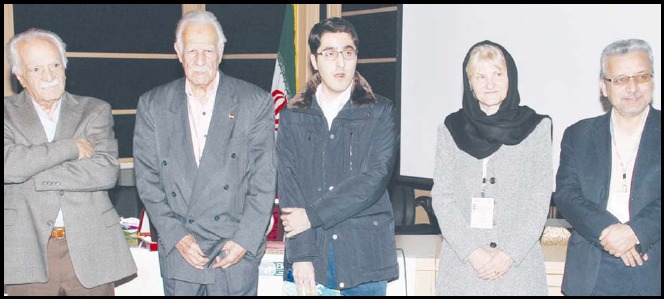
Prof.Karim Vessal from Iran, Prof. Ahmed Said El-Morsy from Egypt and Dr. M.H. Jafary from Pakistan were honoured at Distinguished Editors from the region. Picture taken during this special session shows from (L to R) Prof. Karim Vessal, Prof. Ahmed Said El-Morsy, Mr. Parham Habibzadeh who won the Young Investigator’s Award, Ms Jane Nicholson & Dr. M. Javed Dehghani President ISC.

Speaking at this special session President of EMAME **Prof. Farhad Handjani** said that we should not forget our mentors and role models. This is the first time that we have decided to honour distinguished Medical Editors from this region and he hoped that this tradition will continue at the future conferences as well. It was also for the first time that Young Investigators Award was instituted which was won by Mr. Parham Habibzadeh. He spoke exceptionally well and his topic of presentation was To Cite or Not to Cite Web Sites: an Ongoing Dilemma. The best poster presentation Award was won by Zakavi from Mashad, Iran.

**Dr. Mohammad Reza Ghane** from ISC Iran was the first keynote speaker in the first scientific session jointly chaired by Dr.Fatema Jawad President PAME from Pakistan and Prof. Karim Vessal from Iran. Mr. Reza Ghane’s presentation was on “An overview of Open Access Journals in EMR”.

Tracing the history of Open Access Journals he referred to the initiative taken by Lund University and the Directory of Open Access Journals (DOAJ) which was established in 2003. The advantage of open access is that it ensures more citations and brings more readership. The selection criteria for DOAJ are based on peer review, multidisciplinary, multicounty editorial board, journal’s website. At present Egypt has over five hundred journals in DOAJ, Iran has the second and Pakistan has third place in DOAJ. The number of free articles in DOAJ has been gradually increasing from 2008-2014. About 8.90% of articles from EMAME countries are covered in DOAJ which covers journals in all disciplines but majority of them are in Medicine and Natural sciences. Medical Journals, Mr. Ghane said has a more tendency to open access. EMMR repository has more articles from Egypt, Saudi Arabia, Iran and Pakistan.

Referring to the ISI Thompson database, Mr. Ghane said that Iran was on top with first position followed by Pakistan which stands second; Saudi Arabia has the third ranking while Egypt has the fourth position. The annual growth rate of open access, he said, was about 14%. Mr. Ghane concluded his presentation by stating that open access increases visibility of the journals; ensure more readerships, more citations. Research has its impact as it is cited more, it ensures high Impact Factor. Hence, let us all go for open access but we must be careful about misconduct.

During the discussion **Ms Jane Nicholson** from WHO EMRO remarked that Egypt has more international publishers, hence they do not wish to go for open access. She also referred to delayed or partial open access policy adopted by some journals and it is mentioned on the website of the publishers. However, I believe that all journals should go for open access, she added.

**Mentor is defined as Wise Teacher, Guide, Philosopher, Friend, Advisor & Sponsor- Jose Lapane**

The second keynote presenter in this session was Jose **Florencio Lapena Jr**, President of Asia Pacific Association of Medical Editors (APAME) from Philippines. Topic of his presentation was mentoring and coaching to improve the quality of scientific publications. This was an excellent presentation wherein Jose Lapena gave various definitions by eminent personalities of Mentor which were most comprehensive and covered almost every aspect of the life of the mentees. He pointed out that we all have someone as our mentor. Mentoring is different from coaching.

**Figure:**
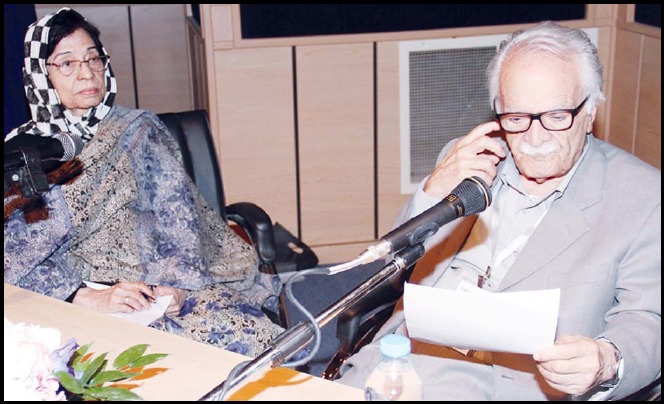
Dr.Fatema Jawad President PAME along with Prof.Vessal from Iran chairing the first scientific session.

Coach helps identify the learning needs but the mentors are guide who lead us on our journey of life, warn us of lurking danger. In fact mentoring is a dynamic relationship. He quoted Don Shula who once said that “I do not know any other way to lead but by example”. A Mentor’s responsibility, Dr. Joseph Lapena said goes beyond that of provider, nurturer or teacher and covers every aspect of one’s life. These include physical, intellectual, spiritual, social and administrative development. A mentor goes even further facilitates the ability to think and act for himself. Another definition of mentor defines it as Wise Teacher, Guide, Philosopher, Friend, Advisor and Sponsor.

Freeman in 1968 defined Mentor as “Someone who is an established practitioner, respected peer who offers through an ongoing professional relationship with his or her mentee opportunities to develop, stimulate and maintain their professional and personal development”. A mentor is the one with whom one can discuss any current professional and personal concerns, who provides space and time to reflect on and evaluate their work. He or she helps to identify further learning needs, offers help and support with personal and professional development. Mentors are also Guides who lead us along the journey of our lives. We trust them because they have been there before. They embody our hopes, cast lighten the way ahead, interprete arcane signs, warn us of lurking dangers and also point out unexpected delights along the way. Mentoring is also a dynamic reciprocal relationship between an advanced career incumbent (the mentor) and a less experienced professional aimed at promoting the development and fulfillment of both. A Mentor also has the remarkable ability not just to give the right answer but more importantly to ask the right questions, the questions which would shift our entire frame of reference. A good Mentor will avoid questions which begin with why but asks questions that requires highest level of thinking. A recall question will give recall answers. Awesome questioning is the most important ability of a Good Mentor. Unfortunately these days, Dr. Lapena said, we have lost the art of listening.

Speaking about the Mentoring process stages, Jose Lapena said that it starts with surrendering which means leveling the playing field both for the Mentor and the Mentee. Second stage consists of accepting which means creating a safe haven for risk taking; Gifting is the main event which is then followed by extending i.e. nurturing the protégée’s independence. Mentoring actions include humility, curiosity and courage, inviting feedback, support and inspiration. Mentoring is a lot different from coaching. Mentoring focuses on an individual as compared to coaching which reflects on performance. A Mentor is a facilitator with no agenda while the Coach has a specific agenda. One is free to select one’s Mentor whereas the Coach comes with a job. A Mentor’s source of influence is on perceived value while the Coach’s source of influence is on position. Mentor’s personal returns consist of affirmation and learning while that of coach’s personal returns are based on teamwork and performance. Mentor’s responsibilities cover the entire life of mentees whereas that of coach is task related. A mentor always focuses on person, their carrier and support for individual growth and maturity. However, the coach is job focused and performance oriented.

Continuing Prof. Jose Lapena said while Mentoring is a power free, two-way mutually beneficial relationship, Mentors are facilitators and teachers allowing the partners to discover their own direction. The coach have a one-way set agenda to reinforce or change skills and behaviours. The coach has objectives, goals for each discussion and session. While self selection is the rule in mentoring relationship with the partner initiating and actively maintaining the relationship but coach comes with the job in an organization. Yet another difference between Mentoring and Coaching, Prof. Jose Lapena said was that while a Mentor’s influence is proportionate to the relationship to the perceived value they can bring to the relationship, a coach has authority by nature of their position, hence ultimately they can insist on their compliance. On the contrary a Mentor has a power free relationship which is based on mutual respect and value. Mentoring relationship is reciprocal there is a learning process for the mentor from the feedback and insight of the partners. However, the coach’s returns are in the form of more team harmony and individual job performance. Coaching even in the sporting arena is task related improvement of knowledge, skills or abilities to better perform a given task.

If one looks at the EMAME objectives, it is also related to Mentoring and all these can be addressed by Mentoring. He advised that everyone should get himself a Mentor and a Friend. Deliberately cultivate professional relationship with such people who spark your creativity and intellectual curiosity. Find mentors who can guide you through the tricky waters of publishing and other elements of professional life. To find a Mentor, one should start from within your department, institution and organization. Then increase your networking at conferences and other events. Make yourself available to peers and senior colleagues to answer questions, brainstorm ideas, proof read manuscripts and provide feedback. They will start to do the same for you. How to select the conferences and events to attend will depend on the availability of resources, travel allowed and the likely benefits of professional networking which will eventually determine your selection. Other issues that might require to be tackled by the researchers, authors will be where to publish, what to publish, what is their institution’s expectations, how much new work is needed and what is the credit given to the published research work by the institution you are affiliated with. It is always worthwhile to send published reports to colleagues and share your accomplishments but refrain from appearing self congratulatory. He concluded his presentation with a quotation from John Henry Newman who said that “To live is to change and to be perfect is to have changed often.”

## PANEL DISCUSSION

This was followed by a panel discussion on Networking and Collaboration to improve the quality of scientific publications. This was moderated by **Prof. Farhad Handjani** while those who participated in the panel discussion included Farrokh Habibzadeh from WAME, Shaukat Ali Jawaid from EMAME, Fatema Jawad from PAME, Behrooz Astaneh from Iranian Society of Medical Editors, Peush Sahni from IAMJE, Jose Lapena from APAME, Wilfred Peh from SAMJE, Elise Langdon-Neuner from EMWA and EASE, Mary Ellen Kerans from MET and Karen Shashok from AuthorAID. It was pointed out that we in this Region are not very much familiar with professional Medical Writers. They have a special place in Medical Writing and it is not only related to Good English only. Ghost authors should work closely with authors. Professional medical writers work for the research scientists, universities, faculty members, pharmaceutical industry. These professional medical writers’ organizations hold their regular meetings, workshops and have a credit system. There is a body of Medical Translators and Interpreters. They work behind the scene; they are not the decision makers. There is no perfect solution to all the problems that we face. We all need to teach and train. We need to talk to each other. ICMJE does not speak for the whole world. Dr.Behrooz Astaneh opined that there is no use of having more and more journals and organizing conferences regularly unless we have a clear roadmap, set agenda and do something constructive and give priority to quality over quantity. We must set some deadlines for the performance of the EMAME and try to accomplish that, he remarked.

Dr. Mohammad Reza Ghani along with Dr. Mohammad Reza Ghani along with Dr. Jehane Tawilah chaired the afternoon session on February 18^th^ the first day of the EMMJ6 Medial Journals conference. **Dr. Wilfred Peh** from Singapore was the first speaker who highlighted the activities of Asia Pacific Association of Medical Editors (APAME). The idea, he said, was conceived during a meeting of WPRIM in Seoul during November 27-28, 2007 and APAME was established at a meeting held at Seoul Korea on May 4-5^th^ 2008. APAME represents 48 countries and upto January 31, 2015 it had one hundred ninety members. We welcome members from other regions as well, he added.

Giving details about the establishments of other associations in the region, he said, Korean Association of Medical Journal Editors (KAMJE) was established in 1996, Malaysian Association of Medial Journal Editors (MAMJE) in 2008, Japanese Association of Medical Journal Editors (JAMJE) in 2008, Mangolian Association of Medical Journal Editors in 2008, Singapore Association of Medical Journals Editors (SAMJE) in 2010. We always try to be inclusive and elect our office bearers of APAME by consensus. WPRIM is a database of the region.

**Dr. Reza Malekzadeh** from Iran speaking in the plenary session chaired by .Prof. Roya Kelishadi along with Dr. Jamshed Akhtar made a presentation on status of science production and innovations in the Middle East and North Africa Region- the challenges and perspective for the future. He pointed out that there are Fifty seven countries of OIC which comprise almost one quarter of the world population but they spend just 2.4% of world’s total expenditures on Research and Development. It also accounts for just 6% of world publications.

OIC, Dr. Malekzadeh said, has an ambitious vision to address this knowledge gap but considerable progress will be required to achieve this. OIC also comprise of some of the richest and some of the poorest countries. There are considerable disparities in terms of scientific and technological output and development within the group. Some of these countries have now started making investment in R&D and it will have impact in future on their development. Some of the wealthiest countries in the OIC still have low levels of R&D. Tunisia, Malaysia, Turkey and Iran spend a lot on R&D. Tunisia, Jordan, Turkey and Iran have the highest number of researchers as a proportion of their population. Turkey and Iran also publish nearly 50% of the OIC publications. Iran has also got the most patents. On an average OIC countries spend 0.46% of their GDP on R&D which represents a modest increase on 2003.

**Educate people, provide them resources, incentives to chase their dreams-Dr.Malekzadeh**

**Figure:**
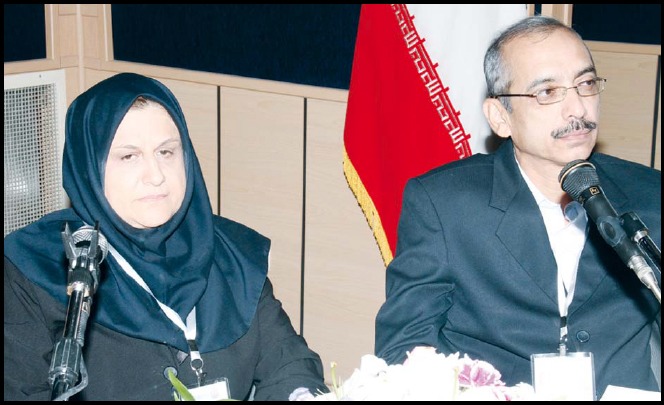
Prof. Roya Kelishadi from Iran along with Dr. Jamshed Akhtar from Pakistan chairing one of the sessions during the EMMJ6 Medical Journals conference held at Shiraz in February 2015.

In higher education, Turkey was on top while Iran has also made significant progress. We are not spending much on R&D and if we take into consideration inflation, actually there is no increase. Main driver behind any innovation process is Human Factor, Technology and Capital. Human factor is the fundamental driver. USA attracts the best people from all over the world and they do interventions. He was of the view that if we educate our people, they will chase their dream. Iran has eighty million population and mostly young people, sixty thousand are enrolled in PhD programme and seventy thousand in medical science universities. Many talented people from China and India are migrating to USA and other developed countries. Majority of the people who make innovations in these countries are immigrants not natives. He made a passionate plea that science and technology needs to be supported at the highest level. OIC countries should foster on equitable social development and high quality research. We should convince our policy makers to invest more in science. OIC has no data to guide their policy makers. Dr. Malekzadeh said the message was clear. In order to build an innovative driven nation we need to educate our people well, provide them enough resources and incentives to chase their dreams, innovations will follow. Iran, he disclosed was the 5^th^ country of origin of migrant inventors in USA. We need more investment in R&D and we also need to protect the creative output of our scientists. Regional journals can play a role in robust peer review systems, merit-based academic career structure, incentives which encourage the commercialization of research. Research evaluation programme should be established and supported across the OIC to ensure that further investment leads to research excellence, Dr. Malekzadeh added.

**Dr. Mohammad Hatem** from WHO EMRO gave details about the medical journals published from the Region and said their number was seventy in 1987 which at present has increased to 598. He also pointed out that not all the journals published in the region are received by WHO and at present IMEMR has 1,53,000 bibliographic citations from the region. Talking about the selection criteria of journals in IMEMR he mentioned peer review, those covering major languages like English, Arabic, French, Farsi and Urdu. English abstract, he said, is mandatory. Egypt is No. 1 followed by Pakistan, Iran, Saudi Arabia and Tunisia as regards the number of manuscripts covered from the region in IMEMR. At present 36% journals covered in IMEMR are from Iran, 24% from Egypt, 13% from Pakistan, Iraq 5% and Saudi Arabia 5%. Out of the total 598 journals at present covered in IMEMR 60% are online which includes 207 from Iran, 67 from Pakistan, 62 from Egypt, and 25 from Saudi Arabia. Of the 598 journals, one hundred fifty five journals are covered by PubMed which is about 26% of the journals published from this region, he added.

**598 journals are covered in IMEMR and 207 of them are also available online-Dr. Mohammad Hatem**

**Dr. Ahmed Mandil** from Eastern Mediterranean Health Journal reviewed the journal’s progress from 1955-2014. WHO, he said, gives research grants in priority areas of public health. We have received thirty one proposals for improving programme implementation through embedded research which are currently under review. From 1995 to 2014, a total of 2704 articles were published in EMHJ. There were about five authors per article on an average. If one looks at the country of first author, Iran is No.1 and Pakistan comes at No.5 as regards the published articles. The least number of articles were published in the subject of emergency preparedness and risks. We need to increase it as we have lot of problems in the region. He also gave details regarding the number of articles published in EMHJ on communicable diseases, non-communicable diseases. About 38% of the studies published had ethics committee approval and another 15.2% had partial approval.

**During the past twenty years, half the manuscripts published in EMHJ came from four countries i.e. Iran, Egypt, Saudi Arabia and Jordan- Dr. Ahmed Mandil**

He concluded his presentation by stating that during the past twenty years, half the manuscripts published in EMHJ came from four countries i.e. Iran, Egypt, Saudi Arabia and Jordan. This calls for further study why other eighteen countries are less represented and how to encourage greater contributions from them. While there were almost equal publications on CD, NCD, HSD, and MCHN (22-26%) more work is needed in the vital field of emergency medicine. EMHJ has to seek prior ethical clearance for research on human subjects as a pre-requisite for considering articles for publication.

During the discussion it was pointed out that authors are reluctant to publish reports on emergency while it is also a cultural issue. It was also stated that manuscripts on emergency medicine are being published in many other journals in the region as well apart from the EMHJ.

There were two parallel workshops on the first day on Journal Metrics, Scientometrics and Altmetrics, Tools for Improving and Increasing Journal’s Visibility and Impact which was conducted by Dr. Payam Kabiri from Tehran University of Medical Sciences Iran and Sustainable Bilingual Journal Publication: The Design of translation and quality control process, including cost considerations by Mary Ellen Kerans from Spain.

## WORKSHOP ON SCIENTOMETRICS

Impact Factor, Payam Kabiri said was the first and the best Scientometrics. However, two year’s window period, he opined, was too short. Secondly IF is also heavily dependent on research field. IF is released during June-July each year. Speaking about citation half life, he said that after some time citations starts decreasing. Citations of Reviews are much better than letters. Citation curve for original papers is more stable. Scopus appeared in 2009 while Elsevier is the second important database. Google Scholar is a new interesting challenger. ISI Thompson/Reuter covers about ten thousand journals while Scopus has thirty thousand journals. Google Scholar has better coverage as it takes data from websites. Most of the researchers use SCOPUS and ISI Thompson for presentations. We also use Google Scholar but Google Scholar cited contents are 2-5 times more. More citations do not mean good quality of research. For example a method is introduced by someone, authors will give his reference and the one who had described that method will continue to get more citations. One should look at the source of citations. For journal evaluation one should look at preference and functions, website, quality, quality of papers published, citations and impact. Reliability and reproducibility is the cornerstone of good indices.

On the other hand, h-Index, Payam Kabiri said takes into account quantity as well as quality of papers. H-Index is median not affected by high number of publications or high number of citations. If all papers get high citation, it improves the h-Index. He then described how to calculate-Index using all the data bases. Retrieved papers also continue to get citations which are not a good activity reflecting current status of the journal. New sciences, new journals, new developments cannot be measured by h-index. Citations take time, atleast five years are needed to get citations. Hence always consider impact of the journal as well not citation alone. SCI mago journal rank considers the prestige of the journal as well. He also talked about SNIP i.e., source normalized impact per paper.

Talking about the social media, he referred to Altmetrics, web of science networking downloads, and view, tweets, blogs and all these have an impact on the society. ALTMETRICS, he clarified, is not academics. Downloads and citations are different. All downloads are not cited. Facilitator in the other workshop on Sustainable bilingual Publications was **Mary Ellen Kerans** from Spain.

**Dr. Peush Sahni** from India spoke about predatory journals. Most of these journals, he said, are waiting for you to submit your manuscripts. Peer review is at the heart of science. Communication has become very fast through internet and e journals. Open access offers the advantages of ease of communication, ease of access and decreased cost. Standards remain the same as per print journals i.e. Submission, peer review and publication. He emphasized the importance of trust, honesty and integrity in scientific publishing. Predatory journals publish all submissions and often inform about the charges after acceptance. Most do not have a peer review system. Even some of them do take money and then do not publish these journals, do not provide any editorial and publishing services, they try to catch unsuspecting and susceptible researchers. These are the journals which are interested in making money. Some open access journals are very strong, have proper peer review system. Predatory journals are smarter, have better websites, appoint guest editors and involve people by making them members of the Editorial Board.

**Figure:**
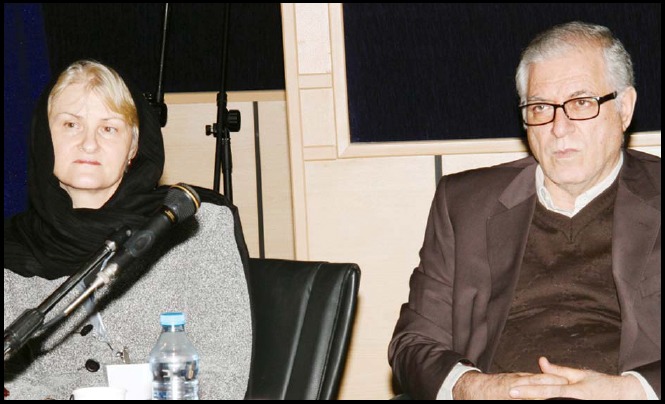
Ms. Jane Nicholson charing one of the scientific session.

Ms Jane Nicholson chaired the next session. **Karen Shashok** from Spain described how to distinguish between legitimate and predatory Open Access Journals. She opined that there are quality and ethical issues not only with open access journals but also with print journals as well. Those who criticize the open access journals are mostly major publishers. It is either the author or the reader who pays to ensure open access. Open Access journal not always ask for authors fees. Paid by authors is known as Golden Open Access and paid by reader is known as Green open access journals. Final version of some papers is immediately available if authors have paid manuscript processing fee and it is known as Hybird model. Journals are both good and bad. She highlighted the importance of transparency and accountability and also referred to the Beal’s list of predatory journals. Those open access journals which are covered either by Directory of Open Access Journals (DOAJ) or are in the Web of Sciences or visible on PubMed maintain their standards and quality. Ideally the journal should identify owners, publishers, editors, editorial board members, should inform location and provide contact information. It should also provide information regarding peer review policy, ethics policy besides giving details about the appeal process.

**Payam Kabiri** in his next presentation talked about quantity and quality of journals published from the Eastern Mediterranean Region. His presentation was based on a survey of published papers in Scopus till 2014. SCOPUS, he said, was an excellent database for physical and biological sciences and has better international coverage. It includes more of Health and Life Sciences contents than other databases. He then displayed the list of top ten journals from the region and five of these were from Pakistan i.e. JPMA, JCPSP, Journal of Ayub Medical College, Pakistan Journal of Nutrition and Pakistan Journal of Medical Sciences. Scopus has 91 journals from Iran, 71 from Pakistan, 82 from Egypt and fifteen from Saudi Arabia. His conclusions were that only 2.8% of 10,067 indexed Health and Life Science journals in Scopus belong to Eastern Mediterranean Region.

**Dr. Ali Akbari Sari** talked about quantity and quality of Iranian Medial Journals. Total number of journals published from Iran, he said, were 1384 of which 137 are in Scopus and 37 are covered in ISI. The number of medical journals published from Iran was ninety in 2005 which increased to 347 in 2015. Of these medical journals, 72 are visible on PubMed, PubMed Central, and Medline while twenty are covered in ISI. All practice peer review, are open access, 95% have English abstracts. One hundred eighty six journals have full text in English, about twenty thousand papers are published by these journals every year and 10% suffer from delayed publication. These journals are published mainly by universities, research centers, professional societies and institutions. Commission for Iranian Medical Journals is based at Ministry of Health and it has nine sub-committee including committee on ethics, technical, websites and biostatistics. The commission controls birth of new journals, helps the journals to improve their quality by providing financial, technical support, training programmes, facilitating in indexing and provision of software’s. The challenges these journals are facing are lack of quality manuscripts, authors usually send their best papers overseas, delay in peer review and lack of professional publishers. Government and national bodies, Ali Akbari Sari stated are playing a positive role providing advice and technical support to medical journals and these challenges need solutions.

In the next session **Dr. Behrooz Astaneh** spoke about metrics of journal quality. Review articles, he said, are highly cited and they inflate the Impact Factor. Self Citations is also used to increase impact factor. Citations to retracted articles are also counted in the Impact Factor. Similarly negative citations also increase IF while different databases have different coverage. Hence, we need to have some measure to rank journals and quality of research, he added.

**Prof. Roya Kelishadi** from Iran spoke about evaluation of Iranian Medical Journals. The request to start a new journal, she said, usually comes from an institution or organization. Then we look for the need and assessment of a new journal and take into account the number of faculty members and students it will serve. We also look at the possibility if it will attract good quality manuscripts in case there are many other journals in the same subject. Then we decide with voting and if approved, they are asked to publish first issue online. It is then evaluated by different committee’s i.e. Ethical, statistical, technical, specialty. We also look at journal website and online management. Annual ranking of the journals, she said, is based on national and international indexing, regular publication, and technical details besides the type of manuscripts published.

**Dr. Jamshed Akhtar** from Pakistan talked about the need for launching a new journal from Pakistan’s perspective. It is the PM&DC which, Dr. Jamshed Akhtar said, recognizes the journals in Pakistan while declaration to publish is given by the Information Department of provincial government. Recently there has been a mushroom growth of biomedical journals in Pakistan. PakMedinet database and the website of the journals were assessed for this study. The study looked at the specialty, frequency of publication, number of original articles per issue and regularity. A total of 69 journals are recognized by PM&DC. These include two monthly, two bimonthly. Thirty four journals were selected for analysis. JPMA published 34 original articles, JCPSP 23, PJMS 41, and PMJ 22. The quarterly journals published JAMC 128 and JPMI 62. University journals JLUMHS published eight articles, JDUHS 16 articles, AKEMU 27 in two issues and Isra University Med J Quarterly 47 articles. He also gave the number of original articles published by specialty organization owned journals. His conclusions were that keeping in view the present number of articles, there was no need to have new journals. He suggested merging the specialty journals, merging regional Associaton journals like EMR Journal of Oncology, EMR Journal of Surgical specialties and EMJR Paediatrics.

**EMAME needs a clear strategic plan with smart objectives which are achievable-Farrokh Habibzadeh**

**Dr. Farrokh Habibzadeh** from Iran spoke about birth control for journals. Faculty members, he said are under pressure to publish for their academic promotions and that is how this problem arises. Almost 450 medical journals are now published from Iran and it is high time that we look at how to stop this mushroom growth of these journals. We should prefer quality over quantity. Selection of papers should be more rigorous. During the discussion Dr. Ali Akbari Sari said that we must go for quality. We need a metrics to sort out these issues. In the Medical Journals Commission we now have some criteria for evaluation of the journals. Dr. Farrokh Habibzadeh suggested changing the rules for faculty members for their promotion adding other yardsticks rather than publications alone.

During the panel discussion on predatory, open access journals it was stated that we should avoid dodgy journals. Look for people who have published in these journals. What is the response and how did you get this response. Predatory journals steal money. Hence one should use wisdom and brain before submitting manuscript to these journals.

On second day of the conference the afternoon session was jointly chaired by Karen Shashok and Mr. Farrokh Saidi. **Mohammad Javed Dehghani** from ISC was the first speaker who in his presentation disclosed that at present the number of journals in ISC database was 3042. It includes 39% in English, 20% in Arabic and the rest 41% are in other languages. In all forty eight countries have sent their journals for coverage in ISC and the number of journals from Pakistan was twenty one. ISC issues the JCR with ranking of journals and Islamic countries universities ranking. ISC, he stated, was ready to help, cooperate and collaborate with every one. We have recently introduced Masters’ programme in Scientometrics, he added.

**Dr. Muhammad Irfan** from Pakistan made a presentation on mutual cooperation among journals, which he opined, was effective in preventing publication misconduct. This was followed by an excellent presentation by **Parham Habibzadeh** from Iran. His title was “To cite or not to cite websites: An ongoing dilemma”. His presentation was based on a study of trend of citation to URLs in five general medical journals from 2006-2013. The journals included were Lancet, BMJ, Archives of Iranian Medicine, Eastern Mediterranean Health Journal, Journal of Postgraduate Medical Institute. They looked at original and review articles, total references and the number of references to URLs. Lancet had 2.9% URL references, BMJ 4.9%, EMHJ 3.7%, AIM 1.4%, and JPMI 1.1%. While 61. 4% websites quoted by Lancet and BMJ were accessible only 42.3% of websites quoted by other journals were accessible. Sometimes if you go to the websites, the referred pages are not found. The solution, he offered was either discourages authors from citing URLs, or archive the cited URLs contents on the journals servers, use only persistent URLs or have some system of internet archiving. He concluded his presentation by stating that there is no universal consensus on preserving the cited URLs though the number of URLs cited is increasing. Only few journals have mentioned in instructions for authors asking authors for archiving the cited URLs.

## GENERAL BODY MEETING

During the GB meeting Dr.M. B. Rokni said that the Executive Committee of the EMAME needs to be more active but it also depends on situation in the region. Prof. Farhad Handjani said that our problems were growing hence we must do something. He suggested that all countries in the region must take up their responsibilities. Iran and Pakistan are doing a lot since we have a will and motivation behind it.

Dr. Behrooz Astaneh from Shiraz was of the view that leadership also matters. We must find out what support the editors need from EMAME and whether they were getting it? We need an action plan with dates and Road Map. Gen. Aslam from Pakistan felt that we need to improve our communication between EMAME and other top bodies in the region and they should be part of our team.

Participating in the discussion Dr. Farrokh Habibzadeh opined that performance of EMAME was not so good and cannot be termed satisfactory. We need a clear strategic plan with smart objectives which are achievable and then we must stick to these objectives. We need some direction and then we must work according to that plan and accomplish that in well defined time. Dr. Akhtar Sherin from Pakistan said let us make EMAME more active. Despite the fact that there are 22 countries in EMAME but in fact it is only two countries Pakistan and Iran which were running the show. We must find out why we are not attracting people from other countries and how many other countries in the EMR region have editor’s societies, he asked. Dr. Irfan said that sub-committees should be more pro active. We must have more activities using information technology, have online discussions, use Webinars and involve others. Dr. Saeed El-Morsy said that we must look at our objectives and then see how we can promote our activities in future.

**EMAME should have an Action Plan with Dates and Road Map - Dr. Behrooz Astaneh**

**Ms Jane Nicholson** pointed out that there was no need to be too much pessimistic. We have achieved a lot ever since the EMAME was founded. We need to be more active and think of some changes. EMAME website is visited quite frequently. WHO EMRO will continue to support the medical journals in the region through different ways. I cannot assure any financial support but we must stay positive, come up with a plan and good proposals which can be looked into.

The first session on Day 3 of the conference was chaired by Mr. Shaukat Ali Jawaid from Pakistan along with Bibi Sedigheh Fazli Bazzaz from Mashad Iran. **Prof. M.B. Rokni** from Tehran was the first speaker who discussed the new challenges in indexing journals in international databases. Prof. Rokni pointed out that in the past inclusion in DOAJ used to take just three four days but now it takes almost nine months. In 2002 he forwarded ten journals cases to ISI of which nine were accepted. During the last four five years the situation has changed a lot. We have to improve our contents and get our journals indexed in one of the important databases of the world. To achieve that one has to follow the criteria they have laid down for indexing. In some journals self citation goes too far which is not advisable. Some journals try to artificially boost their Impact Factor. Our goal is to improve our visibility but we must be careful about the factious Impact Factor. Referring to Scopus, Dr. Rokni said that if your journal is rarely cited, it is a problem. They check the journal editorial board, H. Index and if 30% of the Editorial Board Members have an H. Index of more than three, it is helpful. At least 30% of EDB members should be from overseas. It is also important to add Ethics, malpractice and scientific misconduct statement in the instructions for authors.

**Too much self citation and attempts to artificially boost Impact Factor by journals is not advisable -M.B. Rokni**

**Prof. Farhad Handjani** discussed the ethical issues and conflict of interest as regards sources of revenue for medical journals. Some journals, he said, sell supplements to the Pharma industry to generate revenue. Sale of reprints of published manuscripts is yet another source of revenue for some journals. Advertisements, processing and publication charges is important source of revenue. He was of the view that we must follow the WAME, ICMJE guidelines on advertising. **Pippa Smart** discussed the new journals model i.e. Recorded CD and Video Journals.

**Figure:**
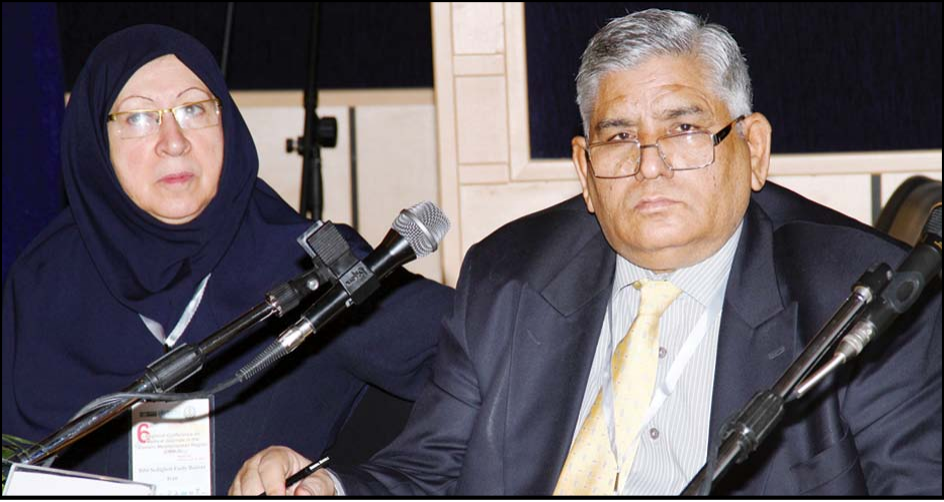
Mr. Shaukat Ali Jawaid GS EMAME alongwith Bibi Sedigheh Fazli Bazzaz from Mashad Iran charing one of the scientific session on Day 3 of the EMMJ6 Conference.

**Prof.Major Gen. Aslam** from Pakistan talked about the attitude towards publications in electronic journals and how the decision makers in the universities look at it. His presentation was based on response received from 290 decision makers from eleven universities in the EMR i.e Pakistan, Iran, Bahrain, Egypt, Saudi Arabia and UAE. Their findings were that at present there is significant prejudice amongst the decision makers in universities in the EMR about articles published in electronic journals. This was mainly due to the lack of knowledge about the quality indicators of electronic publications and it results in un-necessary delay in decision making.

Prof. Maj. Gen. M. Aslam from Pakistan along with Elise Langdon-Neuner chaired the next session. **Mary Ellen Kerans** from Spain spoke about what motivates bilingual journal publishing while **Elise Langdon** made her presentation on “Is there a chance for eloquence in medical articles. During the discussion it was stated that we need well done research which should be well presented. At times the authors are not in a position to convey their message; hence we have to work on the writing side as well. Dr. Behrooz Astaneh opined that not many editors will try to improve the bad English as they are too busy. Mr.Shuakat Ali Jawaid said that though at times we try to help the authors but it is not possible to read the mind of the authors as to what they wish to say and communicate. The authors should be advised to use plain simple English which the readers can easily understand.

In the next session chaired by Ahmad Said El-Morsy along with F. Sharif, **Ms Karen Shashok** from Spain spoke about Editors expectations for Good English. She also highlighted the honorary work she has been doing through Author Aid in Eastern Mediterranean Region. Letters to the Editor, she opined, is post publication review. She also talked about retraction, editorial process to investigate. The journals should clearly define their policy on these issues, she added. **Dr. Fatema Jawad** in her presentation stated that workshops are beneficial in faculty development which was the outcome of two workshops held in Pakistan. **Dr. Ahmad Said El-Morsy** speaking about the art of reviewing emphasized on polite, friendly attitude. Reviewers must devote some time for the job, focus on contents and give suggestions which help the authors to improve their manuscripts.

Dr. M.B.Rokni along with Prof. Sina Aziz chaired the last session. **Mr. Shaukat Ali Jawaid** from Pakistan gave details of a case study related to Citation Amnesia. In his presentation, he also suggested how to face such a situation and highlighted the fact that Editors must be fully aware of their rights as well along with their responsibilities. Giving details of the accomplishments of Author Aid in Eastern Mediterranean Region, **Karen Shashok** pointed out that with the help of her colleagues they edited 376 manuscripts of which 250 were published in 2014. She emphasized that we do not rewrite the manuscripts as we want authors to learn. We cannot check for plagiarism as we do not have the software. We do not check the references as we wish the authors to learn this. We have different levels of expertise, people are working in different areas of research and then there is the question of their availability. Capacity of AuthorAid in editing varies. We provide guidance and advice on specific cases and ask the users to find their own solutions.

The conference concluded with two workshops one conducted by Prof. M.B. Rokni on How to Index your Journal in international indexing systems while the facilitator for the other workshop on Common mistakes in English was Karan Shashok.

